# Evaluation of the mTORC activity in the presence of *Toxoplasma gondii* and azathioprine in human monocyte cell line

**DOI:** 10.1186/s12866-023-02819-8

**Published:** 2023-03-21

**Authors:** Sara Nemati, Hanieh Mohammad Rahimi, Anna Meyfour, Hossein Pazoki, Hamid Asadzadeh Aghdaei, Shabnam Shahrokh, Hamed Mirjalali

**Affiliations:** 1 Foodborne and Waterborne Diseases Research Center, Research Institute for Gastroenterology and Liver Diseases, Shahid Beheshti University of Medical Sciences, Tehran, Iran; 2 Basic and Molecular Epidemiology of Gastrointestinal Disorders Research Center, Research Institute for Gastroenterology and Liver Diseases, Shahid Beheshti University of Medical Sciences, Tehran, Iran; 3 Department of Medical Microbiology, Faculty of Medicine, Infectious Diseases Research Center, Gonabad University of Medical Sciences, Gonabad, Iran; 4 Gastroenterology and Liver Diseases Research Center, Research Institute for Gastroenterology and Liver Diseases, Shahid Beheshti University of Medical Sciences, Tehran, Iran

**Keywords:** *Toxoplasma gondii*, Autophagy, mTORC, Azathioprine, Inflammatory Bowel Diseases

## Abstract

**Background:**

Autophagy is an important part of pathogenesis of IBD. Thiopurines such as azathioprine (AZA) are approved drugs for clinical practices in IBD patients. Besides, as an escape strategy, *Toxoplasma gondii* can use the mTORC1 complex to inactivate autophagy.

**Methods:**

In this study, we investigated whether *T. gondii* tachyzoites may modulate autophagy and interfere the effects of azathioprine in IBD treatment. PMA-activated human monocyte cell line (THP-1) was infected with fresh *T. gondii* RH tachyzoites. After 5 h of infection, the cells were treated with AZA for 6 h. The expression of *atg5, atg7, atg12*, *lc3b*, and *β-actin* (BACT) genes was evaluated using quantitative real-time PCR. To analyze the phosphorylation of ribosomal protein S6 (rpS6), western blot using specific primary antibodies was performed.

**Results:**

The results of real-time PCR revealed that AZA, *T. gondii* tachyzoites, and a combination of AZA and *T. gondii* tachyzoites upregulated *atg5* gene for 4.297-fold (*P*-value = 0.014), 2.49-fold (*P*-value = 0.006), and 4.76-fold (*P*-value = 0.001), respectively. The *atg7* gene showed significant upregulation (2.272-fold; *P*-value = 0.014) and (1.51-fold; *P*-value = 0.020) in AZA and AZA */ T. gondii*, respectively. The expression of *atg12* gene was significantly downregulated in AZA and *T. gondii* tachyzoites for (8.85-fold; *P*-value = 0.004) and (2.005-fold; *P*-value = 0.038), respectively, but upregulated in *T. gondii*/AZA (1.52-fold; *P*-value = 0.037). In addition, the *lc3b* gene was only significantly changed in AZA */ T. gondii* (3.028-fold; *P*-value = 0.001). Western blot analysis showed that *T. gondii* tachyzoites significantly phosphorylated rpS6, and tachyzoites did not interfere the effects of AZA to phosphorylate the rpS6.

**Conclusion:**

Taken together, although AZA and *T. gondii* similarly affects the expression levels of *atg5, atg7*, and *atg12*, but *T. gondii* does not seem to modulate the effects of AZA via mTORC functions.

**Supplementary Information:**

The online version contains supplementary material available at 10.1186/s12866-023-02819-8.

## Background

Autophagy is a cellular process, which scavenges and recycles unused proteins and damaged organelles [1, 2]. Autophagy is significantly involved in the systemic homeostasis of an organism [3]. This process is mainly modulated by AMP-activated protein kinase (AMPK) and mammalian/ mechanistic target of rapamycin (mTOR) [4–6]. The mTOR, as a master regulator of autophagy, activates the ATG1 complex [7], and AMPK (as a significant energy sensor) regulates autophagy via inhibition of mTOR signaling [8]. The mTOR is a member of the phosphatidylinositol 3-kinase (PI3K)-related family, which regulates several cell mechanisms from growth and proliferation to recycling via autophagy [9–11]. However, rapamycin inhibits mTORC1 complex and strongly induces autophagy [12, 13].

Interestingly, autophagy also regulates the immune system and broad-spectrum of inflammatory cytokines [3]. Autophagy plays crucial role in pathogenesis of cardiovascular disease [14], cystic fibrosis (CF) [15], Huntington’s disease [16], cancers, and infectious diseases [3]. In addition, failed autophagy has been linked to numerous diseases, particularly autoimmune disorders, such as systematic lupus erythematosus (SLE) [17], rheumatoid arthritis (RA) [17], experimental autoimmune encephalomyelitis (EAE) [18] and inflammatory bowel diseases (IBD) [19].

IBD is a gastrointestinal disorder, which is mainly categorized to crohn’s disease (CD) and ulcerative colitis (UC) [20]. Regarding the critical role of autophagy in pathogenesis of IBD, modulation of autophagy seems to be a pharmaceutical target for resolution of inflammation during this autoimmune disease [21]. Thiopurines such as azathioprine (AZA), 6-mercaptopurine, and 6-thioguanine, are currently approved drugs for clinical practices in IBD patients [22]. It was shown that thiopurines can activate autophagy by different mechanisms [23]. For example, AZA, as an immunosuppressive agent, not only inhibits purine synthesis in DNA/ RNA of B and T cells [24], but also may downregulate mTORC1, activates autophagy, and controls inflammation [25]. Similar clinical evidence [26, 27] supports the role of sirolimus (rapamycin), an mTORC1 inhibitor, in upregulating autophagy and improving the clinical manifestations of IBD.

*Toxoplasma gondii* is a cosmopolitan parasite that invades, survives, and replicates into any nucleated cell in humans and warm-blooded animals [28]. The evidence has demonstrated that autophagy is critically involved in protection against *T. gondii* in activated macrophage, as well as clearance of inflammation [29]. *T. gondii* employs many strategies to escape from the immune responses. As an escape strategy, *T. gondii* can use the mTORC1 complex and enhances S6K phosphorylation to inactivate autophagy and modulate host immune system [30–32].

Therefore, regarding the high prevalence of toxoplasmosis [33], the role of *T. gondii* in triggering of IBD [34], the crucial role of ATG5, ATG7, and ATG12 in lipidation of LC3-I to form LC3-II in autophagy process during the infection by *T. gondii* [35], and conflicting effects of *T. gondii* and thiopurines on mTORC1, we hypothesized whether *T. gondii* tachyzoites may modulate autophagy and interfere the effects of azathioprine during IBD treatment.

## Methods

### Ethical approval

This project was approved by the Ethical Review Committee of the Research Institute for Gastroenterology and Liver Diseases, Shahid Beheshti University of Medical Sciences, Tehran, Iran (IR.SBMU.RIGLD.REC.1398.032).

### ***T. gondii*** source

Tachyzoites of the virulent RH strain of *T. gondii* were prepared by the *Toxoplasma* lab directed by Dr. Seyed Tabaei (Shahid Beheshti University of Medical Sciences, Tehran, Iran). The harvested tachyzoites from the peritoneal cavity of infected BALB/c were washed with sterile phosphate-buffered saline (PBS; pH = 7.4). In order to evaluate the viability of tachyzoites, samples were stained by trypan blue and counted using a hemocytometer.

### THP-1 cell line

Firstly, THP-1 (human monocyte) cell line (ATCC: TIB-202) was cultivated in 25-cm^2^ culture flasks containing RPMI 1640 medium (Sigma, USA), 10% heat-inactivated fetal bovine serum (FBS; Sigma, USA), and 1% Pen/Strep (1% penicillin/streptomycin), and incubated at 37 °C and 5% CO_2_ atmosphere. To differentiate THP-1 cells to M0 macrophages, 4 × 10^5^ of cells were counted and transferred to a 12-well flat bottom plate, and incubated with 30 ng/ mL of phorbol 12-myristate 13-acetate (PMA; Santa Cruz Biotechnology Cat No. sc-3576) at 37 °C and 5% CO_2_ for 36 h. Macrophage differentiation was microscopically evaluated by an inverted microscopy. Afterwards, supernatant and non-adherent cells were removed, and M0 macrophages were rested together with PMA- and LPS-free cell culture medium (RPMI medium with 10% FBS, without antibiotic) for 24 h at 37 C with 5% CO_2_ [36].

### ***T. gondii*** and Azathioprine Treatment

Azathioprine (Imuran®) 6-(1-Methyl-4-nitroimidazol-5-yl) was in crystallized solid. To prepare the drug, AZA crystals were resolved in dimethyl sulfoxide (M_2_SO) and stored in -20˚ C. M0 macrophages were infected with 4 × 10^5^ (multiplicity of infection [MOI] = 1) of fresh *T. gondii* RH tachyzoites. After 5 h of infection, the cells were co-incubated with 120 µM /mL of AZA for 6 h [25]. To compare the results, two separated wells of M0 macrophages were treated with only 120 µM /mL of AZA and *T. gondii*, respectively. A well of PMA-activated THP-1 without any treatment was considered as control. The treatment durations for AZA and *T. gondii* were adjusted 6 h and 5 h, respectively. All experiments were performed in duplicate.

### Gene expression analysis

Total RNA was extracted from THP-1 cell line in each tested well using total RNA extraction kit (Yekta Tajhiz Azma, Tehran, Iran) in accordance with the manufacturer’s protocol [37]. Extracted RNA was purified by DNase (Thermo Fisher Scientific) treatment, and its concentration was calculated by NanoDrop (NanoDrop Technologies, USA). After adjustment complementary DNA (cDNA) was synthesized using cDNA synthesis kit (Yekta Tajhiz Azma, Tehran, Iran).

To analyze the expression of levels of *atg5, atg7, atg12*, *lc3b*, and *β-actin* (BACT) genes, amplification of corresponded genes was performed using Rotor-Gene Q (Qiagen, Germany) thermocycler. The reaction mixture of 20 µl contained 1 µL of each cDNA sample, 10 µl SYBR green qPCR master mix 2X (Ampliqon, Denmark), and 0.5 µL of each primer [37]. The final volume was adjusted by adding RNase/DNase-free water. The thermal cycling conditions consisted of an initial denaturation of 10 min at 95℃ followed by 40 cycles of 95℃ for 20 s, 59–61 °C for 30 s, 72℃ for 20 s and a final extension step at 72℃ for 20 Sect. [37]. The melt curve analysis was performed for each gene to rule out nonspecific amplifications. Relative expression level of each gene was compared to the *β-actin* gene, and results were analyzed using the 2^− ΔΔCt^ method incorporated into the relative expression software (REST).

### Western blotting analysis

To analyze the presence of rpS6 in experiment wells, total cells were lysed using the cell lysis buffer (Abcam; cat no.ab156034) supplemented with mini EDTA-free protease inhibitor cocktail (Roche, cat no.4693159001). The concentration of THP-1 cells-derived proteins was measured using the BCA protein assay kit (Pars Tous; cat no. A101251). Protein concentration was adjusted and boiled at 100 °C for 5 min in the presence of a loading buffer. Then, equal volumes of protein extract were sperated by 10% sodium dodecyl sulphate-polyacrylamide gel electrophoresis (SDS-PAGE), and then transferred to the polyvinylidene difluoride (PVDF) membrane. The blocking was performed overnight at 4 °C in 5% (wt/vol) bovine serum albumin (BSA) in TBS-T (Tris-buffered saline with Tween 20). Samples were then incubated for 2 h with the specific primary antibodies (rat anti-human rpS6 antibody [R&D Systems; 1:1000, MAB54361-SP], and mouse anti-human/mouse/rat beta-actin antibody [Novus Biologicals; 1:1000; NBP1-47423]. To visualize the presence of proteins, secondary antibodies, goat anti-rat IgG antibody HRP conjugate (Sigma; 1:1000; AP136P) and goat anti-mouse HRP-conjugated antibody (Novus Biologicals; 1:1000; NBP2-30347 H), were employed. Rapamycine was employed as positive control. The expression of the proteins was visualized using the enhanced chemiluminescence detection system (Fusion Solo S, VILBER, France). Protein levels were analyzed with ImageJ software.

## Results

### Quantity analysis of ***atg5, atg7, atg12***, and ***lc3b*** genes

**Fig. 1:**
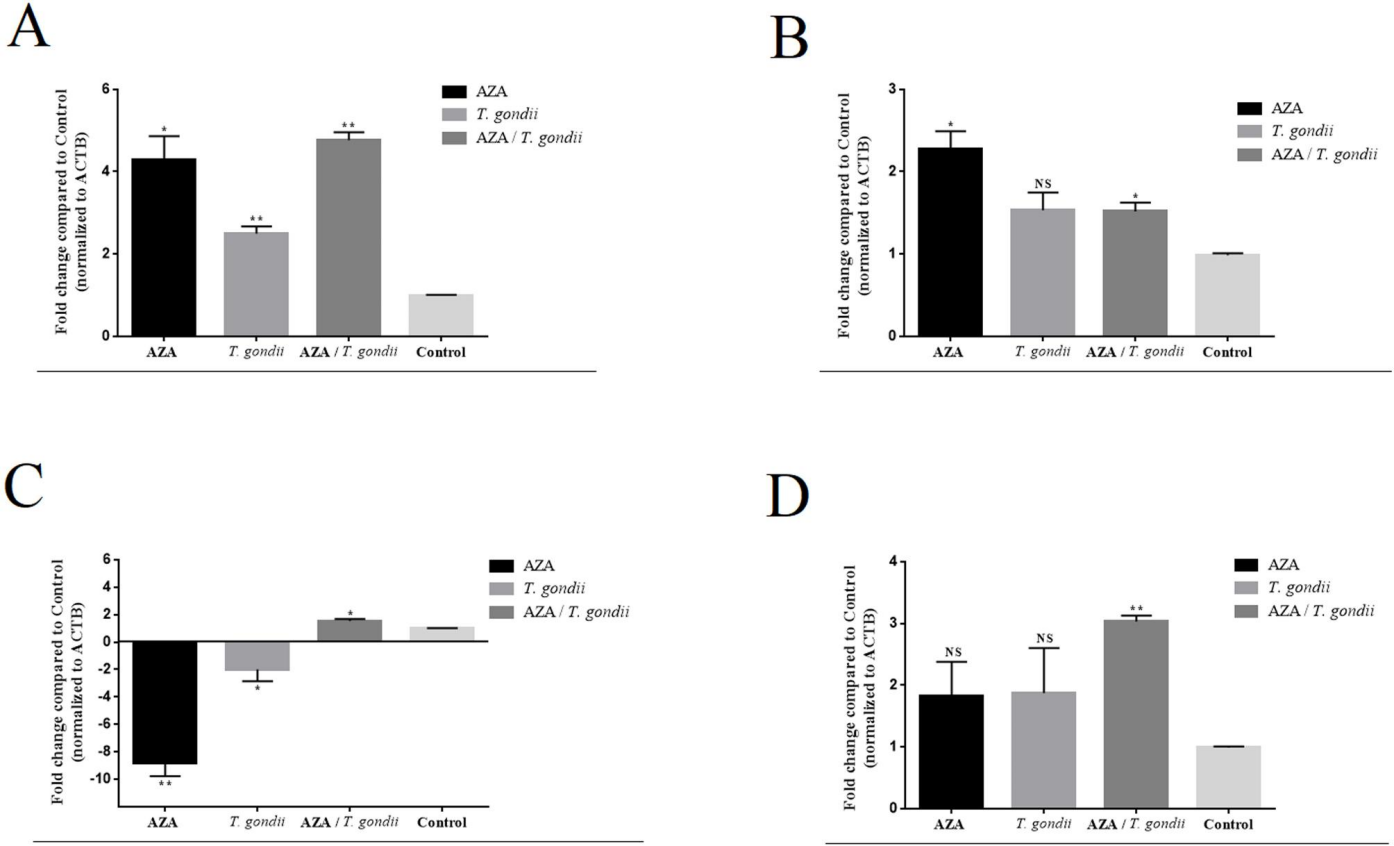
The expression levels of **A) ***atg5*, **B) ***atg7*, **C) ***atg12*, and **D) ***lc3b* genes in THP-1 cell line co-incubated with *T. gondii* tachyzoites and azathioprine. * *P* value < 0.05; ** *P* value < 0.01; NS: not significant. AZA: azathioprine; ACTB: *β-actin*

The results of real-time PCR showed significantly changes of *atg5* gene compared to the control. Accordingly, AZA, *T. gondii* tachyzoites, and combination of AZA and *T. gondii* tachyzoites upregulated *atg5* gene for 4.297-fold (*P*-value = 0.014), 2.49-fold (*P*-value = 0.006), and 4.76-fold (*P*-value = 0.001) compared to the control, respectively (Fig. 1A).

Similar to the *atg5* gene, the expression of *atg7* gene revealed significant upregulation in THP-1 cells treated by AZA (2.272-fold; *P*-value = 0.014) and AZA */ T. gondii* (1.51-fold; *P*-value = 0.020) compared to the control, while *T. gondii* tachyzoites did not induce the expression of *atg7* gene (Fig. 1B).

The *atg12* gene was significantly downregulated in AZA (8.85-fold downregulation; *P*-value = 0.004) and *T. gondii* tachyzoites (2.005-fold; *P*-value = 0.038) and upregulated in *T. gondii*/AZA (1.52-fold; *P*-value = 0.037) (Fig. 1C). Furthermore, the expression changes for *lc3b* gene was only significant in M0 macrophage cell line cured by AZA */ T. gondii* (3.028-fold; *P*-value = 0.001), compared to the control (Fig. 1D).

**Fig. 2:**
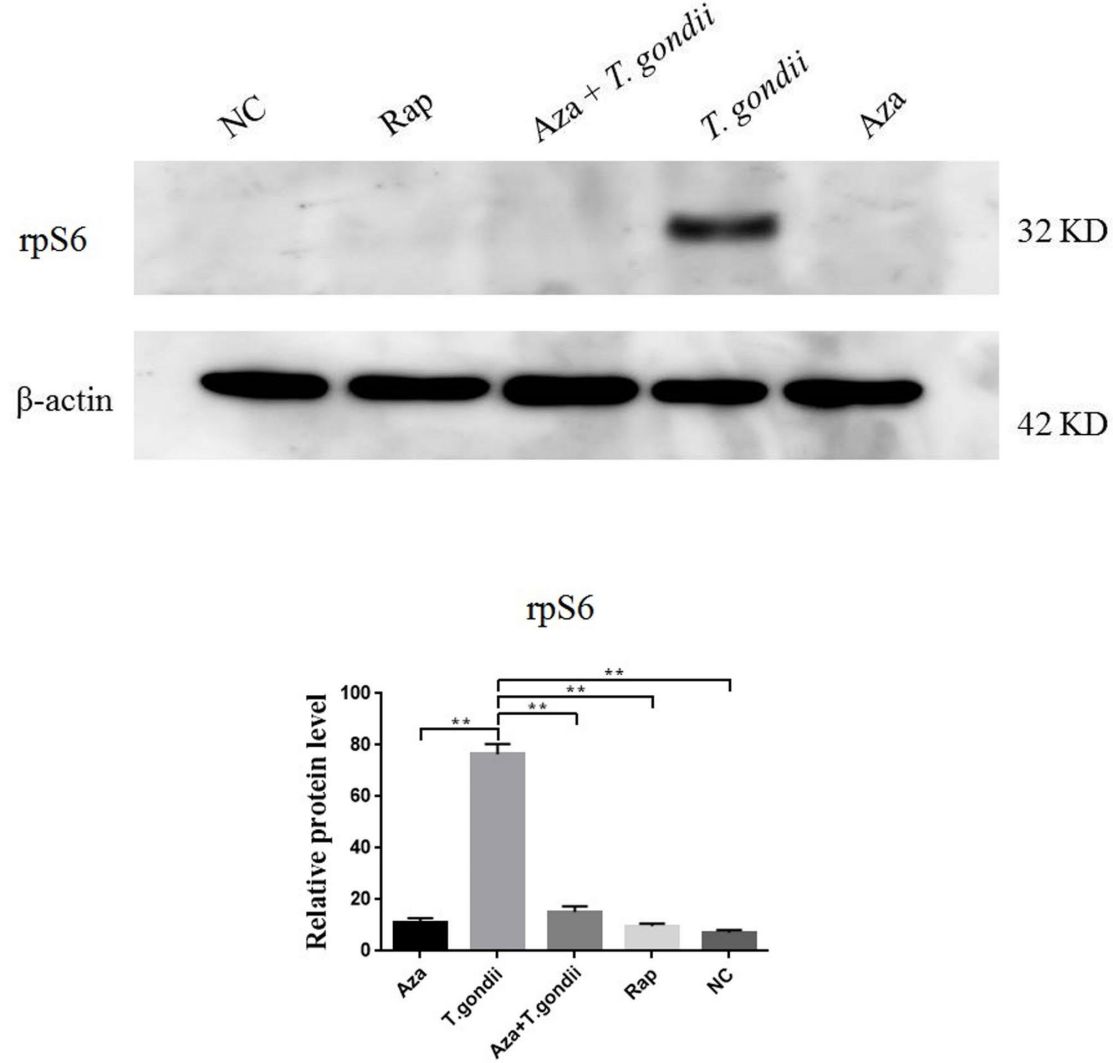
The westernblot analysis shows phosphorylation of rpS6 by *T. gondii* to activate mTORC and prevent autophagy formation. Dephosporylation of rpS6 at the protein level by azathioprine suggests the similar effects of azathioprine and rapamycin in activating autophagy. * *P* value < 0.05; ** *P* value < 0.01. AZA: azathioprine; Rap: rapamycin; rpS6: ribosomal protein S6; ACTB: β-actin; NC: negative control

Western blot analysis showed that *T. gondii* tachyzoites were significantly phosphorylated rpS6, while AZA did not phosphorylate the rpS6, as expected. In addition, *T. gondii* tachyzoites did not interfere the effects of AZA to phosphorylate the rpS6 (Fig. 2).

## Discussion

Autophagy pathway is modulated by the ATG proteins, which are initiated by vesicle nucleation. LC3/ATG8 and ATG12 are ubiquitin-like molecules, which plays central role in autophagosome biogenesis [37, 38].

Autophagy is able to directly participate in immune cell functions through regulating the secretion of cytokines and chemokines [39, 40]. Autophagy may regulate the IL-1β and IL-23 secretion to protect from autoimmune diseases [41–43]. Interestingly, polymorphisms through the *il-1β* and *il-23* genes may lead to susceptibility to IBD [41, 44]. Additionally, autophagy-related protein 16 − 1 (ATG16L1), nucleotide-binding oligomerization domain-containing protein 2 [NOD2], immunity-related GTPase family M protein [IRGM], and leucine rich repeat kinase 2 [LRRK2] have been suggested as genetic susceptibility loci for CD based on genome-wide association studies (GWAS) [45–47]. Wittkopf et al. [48], showed that although granule formation of paneth cells was affected in ATG7-knock out mice, but depletion of only ATG7 did not disturb the immune homeostasis. Regarding the promising role of autophagy in control of IBD symptoms, modulation of canonical autophagy pathway via mTOR seems to be a treatment approach for patients with IBD [49, 50]. The role of sirolimus (rapamycin), as mTOR inhibitor and autophagy activator, was clinically investigated in amelioration of the IBD symptoms [27, 51]. In a case study, Massey et al., [27] evaluated the effects of sirolimus on a 37-year-old woman with refractory colonic and perianal CD for six months and reported a remarkable improvement in progress of the disease. Mutalib et al. [26], evaluated the effects of sirolimus in IBD patients (UC and CD), and suggested that inhibiting mTORC using sirolimus could be considered a rescue therapy, particularly in children with severe IBD refractory. The results of an experimental study by Hu et al., [52] in mice model supported previous findings and documented that inhibiting mTORC attenuates dextran sulfate sodium (DSS)-induced colitis. In addition, a single center study on 15 CD-related fibrotic stricture patients by Zhong et al. [53], demonstrated favorable effect of rapamycin in symptoms of IBD patients with CD-related stricture in upper gastrointestinal tract. Hooper et al., [25] demonstrated that AZA not only modulates the immune responses during the IBD onset, but also induces autophagy via inhibiting mTORC1. In the line of these studies, our findings showed that AZA induced the expression of *atg5* and *atg7*, which are essential for formation of the autophagy complex [54, 55]. In addition, protein analysis showed that AZA induces dephosphorylation of rpS6 and subsequently autophagy progress.

The AZA is an immunosuppressant, which its role in immunomodulation during immune related diseases has well been established [56, 57]. The impact of azathioprine-associated lymphopenia on opportunistic infections was investigated that the findings showed lack of significant upper incidence of the infections compared to control group [58]. However, prescription of additional immunomodulation agents increased the risk of opportunistic infections in IBD patients [58]. Therefore, re-activation of *T. gondii* tachyzoites in IBD patients could be an important challenge. *T. gondii* employs strategies to survive and replicate inside host cells via avoiding from phagolysosome formation. Wang et al. [32] investigated the effects of *T. gondii* on host mTOR signaling and showed that *T. gondii* induces mTOR-dependent cell cycle progression and cell growth. Actually, *T. gondii* modulates mTORC and suppresses autophagy [59]. Notable, as an escape strategy, *T. gondii*, type I and II strain, may induce Akt activation followed by triggering epidermal growth factor (EGF), which results in mTORC phosphorylation and autophagy negative regulation [60]. This fact may explain the lack of expression in LC3 transcription in THP-1 cell line infected by *T. gondii* RH strain. Our findings represented that *T. gondii* in THP-1 cell downregulated transcription of *atg*12, while significant phosphorylation of rpS6 in protein level, in the line of previous studies, proposes deregulation of autophagy via mTORC manipulation by *T. gondii* [61, 62]. However, our finding suggests that although immunosuppressant medications prescribed in IBD condition can increase the risk of opportunistic infection and likely re-activation of latent toxoplasmosis, *T. gondii* tachyzoites are not able to phosphorylate mTORC and inhibit autophagy during AZA treatment in IBD patients.

The upregulation of *atg5, atg7, atg12*, and *lc3b* genes transcription in THP-1 cell line treated by both AZA and *T. gondii* tachyzoites was reported by our results. Actually, formation ATG5-ATG12 and ATG16 is critical for lipidation of LC3-I and establishment of LC3-II [63]. As finding, our results showed an increase in the expression level of *atg5* gene. This finding is supported by previous studies indication upregulation of *atg5* gene or its protein product upon invasion of *T. gondii* [64, 65]. However, the lack of significant upregulation of *atg7* and *lc3b*, and downregulation of *atg12* genes are mostly time-dependent. In this regard, Wang et al. [64], proposed higher production of *lc3b* gene after 24 h compared to 8, 4, and 2 h post-infection. The inconsistency between higher expression of *atg5* in THP-1 cell line treated by *T. gondii* with rpS6 phosphorylation may support finding released by Wang et al. [64], indicating mTOR-independent autophagosome recruitment. In addition, in the current study the expression of ATG-related genes was investigated in transcription level and considering the lack of protein analysis, these results should be carefully interpreted. Interestingly, our finding showed that co-existence of AZA and *T. gondii* synergistically increase the expression of all studied ATG-related genes. As mentioned above, AZA increases the expression of ATG-related genes and induces dephosphorylation of rpS6 to provoke autophagy process.

Moreover, in the current study, we employed strain I, which is not suitable candidate to study reactivation of *T. gondii*; however, this strain is popular genotype, which has been investigated in autophagy studies [64, 66, 67]. Nevertheless, there is no data investigating the bilateral effects of *T. gondii* and AZA. Therefore, in vivo study of this bilateral effect of AZA and *T. gondii*, investigation of the communication between different strains (I, II, and III) and autophagy, regarding prescription of AZA, and repeating this study in different timepoints and cell lines could be an interesting field of study.

## Conclusion

This study showed that *T. gondii* tachyzoites significantly phosphorylated rpS6, while they were not able to obstruct the autophagy activation by AZA treatment. Taken together, simultaneous treatment of THP-1 cell line by both *T. gondii* tachyzoites and AZA showed overcoming the effects of AZA in activation of autophagy. However, this is a preliminary study and further investigations in animal models and with different strains of *T. gondii* are needed to rule out the negative effect of *T. gondii* tachyzoites on autophagy activation upon prescription of AZA.

## Electronic supplementary material

Below is the link to the electronic supplementary material.

Supplementary Material 1

## Data Availability

All data generated or analyzed during this study are included in this published article [and its supplementary information files].
