# Supplementary material for: Evaluation of the mTORC activity in the presence of Toxoplasma gondii and azathioprine in human monocyte cell line
Source: BMC Microbiol. 2023 Mar 21;23:77. doi: 10.1186/s12866-023-02819-8 (PMC10029279; doi:10.1186/s12866-023-02819-8)
Supplement: Supplementary file 1 — Supplementary Material 1 [file 12866_2023_2819_MOESM1_ESM.pdf]

Toxoplasma gondii Modulates mTORC, but does not Interfere the Effects of Azathioprine Prescribed in IBD Conditions

Sara Nemati [1], Hanieh Mohammad Rahimi[1], Anna Meyfour[2], Hossein Pazoki[3], Hamid Asadzadeh Aghdaei[2], Shabnam Shahrokh[4], Hamed Mirjalali[1]

1. Foodborne and Waterborne Diseases Research Center, Research Institute for Gastroenterology and Liver Diseases, Shahid Beheshti University of Medical Sciences, Tehran, Iran
2. Basic and Molecular Epidemiology of Gastrointestinal Disorders Research Center, Research Institute for Gastroenterology and Liver Diseases, Shahid Beheshti University of Medical Sciences, Tehran, Iran
3. Department of Parasitology, Faculty of Medicine, Gonabad University of Medical Sciences, Gonabad, Iran
4. Gastroenterology and Liver Diseases Research Center, Research Institute for Gastroenterology and Liver Diseases, Shahid Beheshti University of Medical Sciences, Tehran, Iran

*****Corresponding Authors:

1. Dr. Hamed Mirjalali; Foodborne and Waterborne Diseases Research Center, Research Institute for Gastroenterology and Liver Diseases, Shahid Beheshti University of Medical Sciences, Tehran, Iran. Email: hamedmirjalali@sbmu.ac.ir, hamed_mirjalali@hotmail.com

ORCID: Hamed Mirjalali; http://orcid.org/0000-0002-2568-902

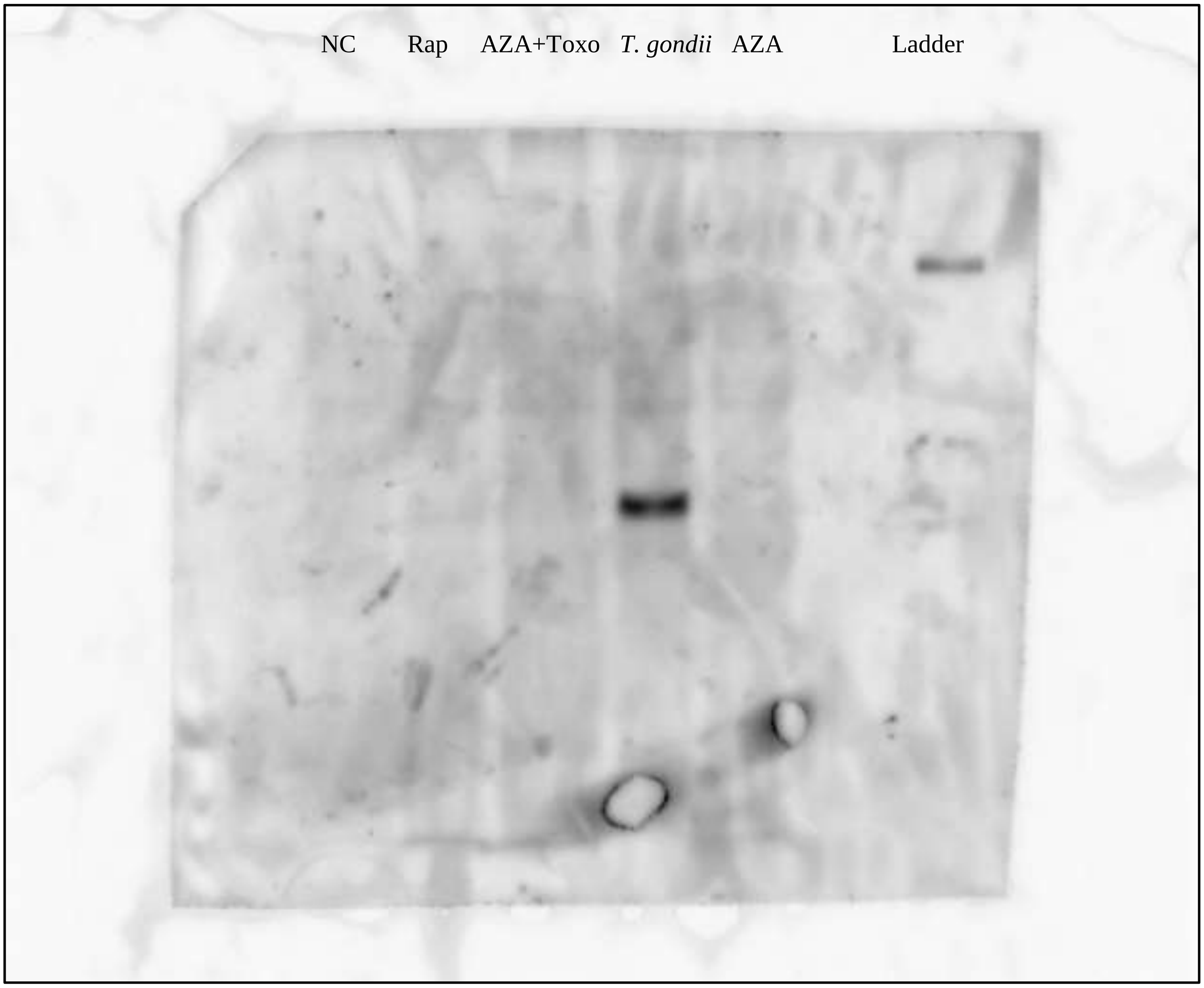

Suppl Fig 1A. Whole blotting without editing. The blot merged with the pS6 signal and marker.

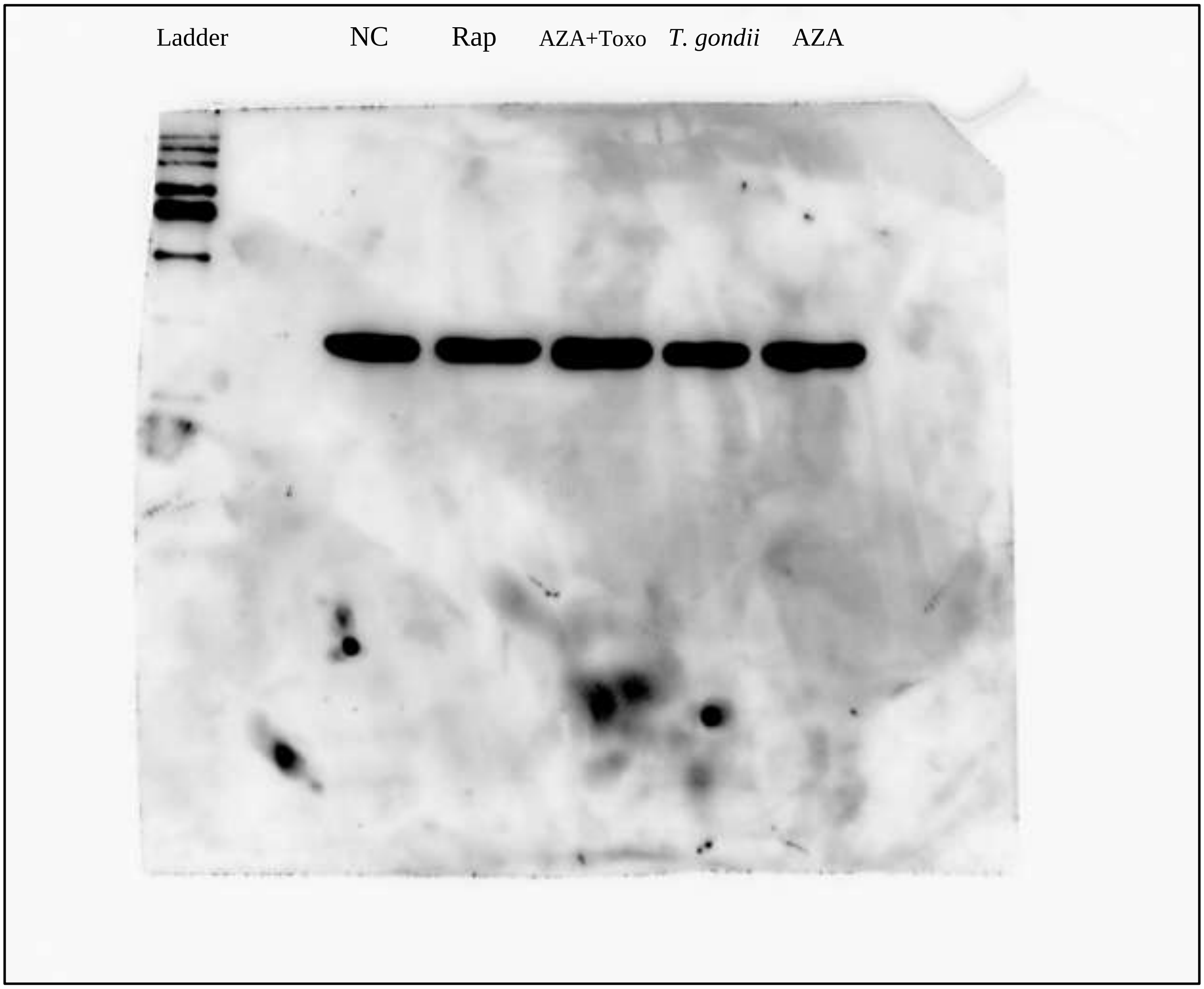

Suppl Fig 1B. Whole blotting without editing. The blot merged with the β-actin signal and marker.
